# An exploratory evaluation of the acceptability of revised physician examinations in South Africa initiated during the coronavirus disease 2019 pandemic

**DOI:** 10.4102/jcmsa.v4i1.388

**Published:** 2026-07-28

**Authors:** Farah Dawood, Veena S. Singaram, Bilkish Cassim

**Affiliations:** 1 Department of Internal Medicine, College of Health Sciences, University of KwaZulu-Natal, Durban, South Africa; 2 Health Professions Education Unit, College of Health Sciences, University of KwaZulu-Natal, Durban, South Africa; 3 Department of Geriatrics, College of Health Sciences, University of KwaZulu-Natal, Durban, South Africa

**Keywords:** physician exit examination, medical education, postgraduate assessment models, South Africa, competency-based assessment, clinical competence

## Abstract

**Background:**

This study explored the acceptability of the decentralised assessment of clinical competency (AOCC) and digital structured oral examination (SOE), introduced in South Africa (SA) during the coronavirus disease 2019 (COVID-19) pandemic, with a view to informing their potential continuation and refinement in post-pandemic contexts.

**Methods:**

A mixed-methods study was conducted with candidates and examiners from SA training centres who participated in the AOCC and SOE in 2020–2021. Quantitative data explored perceptions regarding the credibility of the revised formats. For deeper insights, focus group discussions were held. Data were analysed using descriptive statistics and thematic analysis.

**Results:**

Forty candidates completed the questionnaires with at least one representative from each SA training centre. Twelve examiners responded to the AOCC and 13 to the SOE questionnaires. Both groups viewed the revised formats broadly positively, highlighting the AOCC’s fairness, relevance and acceptability. Analysis highlighted advantages such as logistical benefits, and challenges, including the need for standardisation of registrar training. The SOE was endorsed by more than 70% of participants for its standardisation, objectivity and breadth of content coverage. Both groups expressed concerns about limited examiner–candidate interaction, while candidates reported uncertainty regarding the depth of responses required.

**Conclusion:**

The revised formats were broadly acceptable and perceived as valid alternatives to the previous format of the examination. These findings support the continuation of hybrid, decentralised formats for future assessments.

**Contribution:**

This study provides insight into the benefits and drawbacks of the amended Fellowship of the College of Physicians (SA) examination format, offering guidance for future reforms in medical assessment in SA and comparable resource-constrained contexts.

## Introduction

Training programmes, assessments and examinations for physicians vary globally and are continually evolving in response to modern educational principles and healthcare needs.^1,2,3,4,5^ These changes aim to improve the validity, reliability and alignment of examinations with real-world clinical practice.^1,2,3,4,5,6^ Validity and reliability of assessments are fundamental to producing defensible judgements about professional competence that are credible, reproducible and appropriate to the intended purpose. In high-stakes examinations, these are particularly important to support decisions about readiness for independent clinical practice while maintaining consistency across candidates, contexts and examiners.^6^ Common to all examinations is the need to confirm clinical competency and applied theoretical knowledge.^1,2,3,4,5,6^ Significant changes have been introduced internationally.^1,2,3,4,5^ In the United Kingdom (UK), the long-standing five-station clinical carousel was remodelled with altered station composition and timing to enhance reliability and reduce station-specific bias.^1,2^ These changes, originally scheduled for 2020 but delayed because of the coronavirus disease 2019 (COVID-19) pandemic, were implemented in 2023. In Canada, the written component was moved earlier in training, with emphasis on aligning the examinations with unsupervised practice in a competence by design (CBD) model^3,4,5^ in a multi-year phased implementation from 2015 to 2022. Competency-based medical education (CBME) frameworks, such as Canada’s CBD model, emphasise the longitudinal development of competence through staged training outcomes supported by continuous formative and workplace-based assessment (WBA).^3,4,5^ These shifts reflect a move away from time-based progression towards earlier verification of foundational knowledge, allowing subsequent training to emphasise applied competence, WBA and individualised learning trajectories.

In South Africa (SA), the Colleges of Medicine of South Africa (CMSA) is the unitary examination body for all specialist examinations. Prior to the COVID-19 pandemic, the Fellowship of the College of Physicians (FCP) Part II examinations were held biannually with a written theory component, and a bedside clinical assessment (one long case and two short cases) held at a single venue.^7^

The CMSA had started a process of moving towards CBME, and the process of engagement with trainers had been initiated.^8^

The COVID-19 pandemic had a significant impact worldwide, with restrictions on all gatherings, including those for examinations. The need for physical distancing during the pandemic necessitated the rapid adoption of alternative approaches to the training and examination of undergraduate and postgraduate medical students. In response, many institutions transitioned to remote or modified assessment formats, including online examinations. In addition, the pandemic led to a reduction in non-urgent patient admissions to reduce exposure of potentially vulnerable patients to Severe Acute Respiratory Syndrome Coronavirus 2 (SARS-CoV-2), limiting the availability of patients for training and clinical examinations.

In SA, following the declaration of the National State of Disaster on 15 March 2020,^9^ gatherings and interprovincial travel were restricted. This posed a challenge for the CMSA as conducting the conventional examinations would have meant the convergence of numerous candidates, examiners and recruited patients at a single examination venue. These unprecedented circumstances served as a catalyst for a change in examination formats.

For the FCP Part II, the College of Physicians (CoP) introduced a decentralised assessment of clinical competency (AOCC), to be held at each training site and a national online structured oral examination (SOE). These initiatives were in line with the planned evolution of the clinical examinations and continue in SA postgraduate physician examinations presently.

Although literature exists on pandemic-driven adaptations to medical education and assessments in high-income countries,^10,11,12,13^ there are limited published data on how physician examinations were modified in low- and middle-income countries, particularly in sub-Saharan Africa.

Two studies explored the feasibility and acceptability of SOEs in SA. In a multi-specialty cohort, Burch et al.^14^ conducted a quantitative survey and reported high levels of acceptability and logistical efficiency. While Burch et al. explored trainee perceptions of pandemic-related assessment disruption, their study did not explore candidates’ lived experiences of high-stake postgraduate clinical oral examinations in depth.

In contrast, Lahri and Meyer,^15^ in a qualitative study on SOEs in emergency medicine, found that while registrars and examiners recognised the value of the format, concerns were raised regarding misalignment with clinical reasoning, heightened anxiety, examiner familiarity and language barriers for non-native English speakers.

While these studies offer valuable insights into the acceptability and operational feasibility of web-based SOEs, neither explicitly examined the AOCC, a key innovation introduced by the CoP during the COVID-19 pandemic.

Hence, this study aimed to evaluate the acceptability of two novel assessment formats, the decentralised AOCC and the SOE, introduced for the FCP. It further explores perceived fairness, preparedness and relevance under pandemic-altered conditions, as well as how contextual training experiences shaped candidates’ perceptions of assessment.

## Research methods and design

### Study design

A mixed-methods design was employed to integrate quantitative measures of acceptability with qualitative insights into participants’ experiences and contextual perspectives.

The quantitative component enabled a broad assessment of acceptability, fairness, validity and feasibility across multiple training centres, allowing for an appraisal of the level of support for the revised assessment formats within a national cohort. However, assessment acceptability is a complex, context-dependent construct that cannot be fully captured through survey data alone. The qualitative component therefore complemented these findings by eliciting in-depth accounts of candidates’ and examiners’ lived experiences, enabling exploration of underlying reasons, contextual influences and perceived consequences of the revised formats. Integrating quantitative breadth with qualitative depth enhanced the credibility, depth and interpretive richness of the findings.

The survey questionnaires and focus group discussion (FGD) guides were purpose-designed through an iterative process, guided by the study aims and aligned with key domains of the AOCC and SOE. In the context of the COVID-19 pandemic, online surveys and FGDs were used to enable broad participation while minimising physical contact.

Instruments were reviewed and refined by the investigators, with face and content validity established through expert review and consultation with colleagues external to the study.

A formal pilot was not conducted because of pandemic-related constraints; however, iterative refinement ensured clarity and relevance.

### Setting

To qualify as a physician in SA, registrars must pass the FCP(SA) Part I examination, complete a 4-year clinical training programme and a research component and pass the FCP(SA) Part II examination at exit. The FCP(SA) Part I examination assesses knowledge of the basic sciences and pathophysiology of common clinical conditions in a multiple-choice question (MCQ) format.^7^ The 4-year clinical rotation in accredited facilities includes exposure to general medicine and the core subspecialties of internal medicine.

While the CoP has a recommended curriculum and exit outcomes, the training experience is influenced by the local environment and in many instances, ‘the apprentice’ model is still used. The candidate maintains a portfolio of their training and expertise in relevant procedures. Their performance is assessed after each rotation, and training is signed off by either the head of department or the postgraduate educational adviser.

After 30 months of clinical training, the trainee is eligible for the FCP Part II examination. The theory component consists of an MCQ paper and a written objective test, which includes the interpretation of clinical scenarios, laboratory and imaging results. The candidate needs to pass this component to proceed to the bedside clinical assessment, which assesses the ability to obtain a history, perform a clinical examination, make a plausible clinical assessment and discuss the appropriate investigation and management.^7^

The clinical examination was previously conducted at a single examination centre nationally on a rotational basis. The examination consisted of three clinical cases: one long and two short. Candidates were required to pass two of the three cases and to score ≥ 50% overall.

### Examination context

To maintain the clinical component of the examination during the pandemic, the CoP introduced the AOCC, held at each training centre with strict infection prevention and control measures in place. The examination panel and moderators were local physicians. If a centre had insufficient examiners, examiners from another centre were linked in via Zoom.

In the AOCC, to decrease exposure time, candidates were examined on three short cases only at the bedside and were required to pass two out of the three cases.^16^ Candidates who passed only one out of three cases were assessed on an additional two cases and were required to pass three of the five cases in total. Candidates who failed all three initial cases or three of the five cases were deemed to be unsuccessful and could not proceed to the next component of the examination, the national online SOE, conducted on Zoom 5 –6 weeks later. The SOE consisted of six predetermined clinical scenarios, requiring clinical evaluation, interpretation of investigations and management.

### Quantitative study

Questionnaires were developed to obtain demographic data of participants and to determine their opinions of the AOCC and SOE (Online Appendix 1 and Online Appendix 2). The candidate questionnaire was a single questionnaire with separate sections based on the AOCC and SOE. The examiner questionnaires (AOCC, SOE, or both) were sent separately, as not all examiners participated in both components. The questionnaires included dichotomous, multiple-choice, Likert-scale and open-ended items to capture participants’ experiences of the examination and suggestions for future examinations. Candidates and examiners were invited to participate in the study via the online platform SurveyMonkey, distributed by the CMSA in 2021. Responses to the survey were collated, and the data were managed in Microsoft Excel.

#### Statistical analysis

Frequencies were calculated for dichotomous and multiple-choice answers, and open-ended responses were analysed qualitatively. Responses were categorised into relevant themes, and the perceptions of candidates and examiners were compared to draw insights. The analysis also aimed to uncover nuanced opinions and suggestions that could inform potential refinements in future examinations.

### Qualitative study

Focus group discussions were conducted with all candidates and examiners who accepted an invitation to participate in the study (Online Appendix 3 and Online Appendix 4). Invitations were sent by the CMSA to all eligible candidates and examiners via electronic mail. The discussions were led by the principal investigator, with the co-investigators serving as moderators. Discussions with candidates explored their experience with the AOCC and SOE, as well as the perceived advantages, disadvantages and challenges. Discussions with the examiners explored their perceptions of the examination and how these perceptions could influence the format of future FCP Part II examinations. Prompt questions were used to guide discussions and gather information relevant to the study’s aims and objectives. The discussions were recorded and transcribed verbatim, ensuring the participants’ confidentiality through de-identification of transcripts.

#### Qualitative data analysis

Qualitative data were analysed using reflexive thematic analysis as described by Braun and Clarke.^17^ This approach was selected to facilitate an in-depth exploration of participants’ experiences and perceptions of the revised examination formats, enabling the identification of patterned meanings across the dataset rather than purely descriptive categorisation.

Analysis was conducted iteratively and reflexively. Transcripts were read multiple times to ensure familiarisation with the data. Initial codes were generated inductively through close, line-by-line engagement with the transcripts, capturing both semantic content and underlying meanings. Coding was undertaken as an interpretive process, shaped by the researchers’ perspectives and engagement with the data.

Codes were then actively organised into candidate themes, which were reviewed and refined through ongoing discussion among the research team. Coded extracts were examined for coherence within themes and clear distinctions between themes, with themes refined, collapsed or restructured to enhance conceptual clarity and analytic depth.

In the final stages, themes were defined and named to reflect their central organising concepts, moving beyond descriptive summaries to interpret patterns of meaning across participants’ accounts. The analytic narrative was developed through the integration of these themes with illustrative data extracts and relevant literature.

#### Reflexivity

Researcher reflexivity ensures that the researchers consistently recognise the assumptions they introduce into the study. The authors come from varied disciplinary backgrounds and had distinct educational roles and levels of engagement with the examination process. Farah Dawood was a registrar in training, Bilkish Cassim is an Associate Professor in Internal Medicine and an examiner, Veena S. Singaram is a medical educationalist and researcher with no direct involvement in the registrar training and examination. Although the lead author was an FCP registrar at the time, she did not participate in the examinations studied, and reflexive thematic analysis was conducted inductively, with themes actively generated through analytic engagement with the data rather than determined a priori. Ongoing in-depth discussions throughout the study enabled the team to acknowledge and harmonise these varied viewpoints, thereby enhancing the depth, credibility and richness of the interpretation of participants’ accounts.

### Ethical considerations

Ethical clearance to conduct this study was obtained from the University of Kwazulu-Natal and the Biomedical Research Ethics Committee (No. BREC/00002518/2021). Written informed consent was obtained electronically from all participants. All identifying data were anonymised.

## Results

### Participant characteristics

For the quantitative component, questionnaires were sent to 267 candidates and 74 examiners. Forty candidates and 13 examiners completed the questionnaires, giving a response rate of 15% and 17.6%, respectively. The demographic characteristics of the participants and their university affiliations are shown in Table 1 and Table 2. For the candidate group, there was at least one representative from each of the training centres in SA. Examiners from six of the nine training centres completed the questionnaires, 12 on the AOCC and 13 on the SOE.

**TABLE 1:** Characteristics of candidates.

Demographics	*n*	%
**Respondents to questionnaires**		
AOCC	40	-
SOE	36	-
**Sex**		
Female	13	32.5
Male	27	67.0
**Age group (years)**		
30–34	19	47.5
35–40	10	25.0
> 40	11	27.5
**University**		
Sefako Makgatho Health Sciences University	3	7.5
Stellenbosch University	4	10.0
University of Cape Town	4	10.0
University of Free State	4	10.0
University of KwaZulu-Natal	7	17.5
University of Pretoria	2	5.0
University of Witwatersrand	10	25.0
Walter Sisulu University	4	10.0
Unspecified	2	5.0

AOCC, assessment of clinical competency; SOE, structured oral examination.

**TABLE 2:** Characteristics of examiners.

Demographics	Clinical		SOE	
	*n*	%	*n*	%
Respondents to questionnaires	12	-	13	-
**University affiliation**				
Sefako Makgatho Health Sciences University	1	8.3	1	7.7
Stellenbosch University	5	41.7	5	38.5
University of Cape Town	2	16.7	2	15.4
University of Free State	2	16.7	2	15.4
University of KwaZulu-Natal	1	8.3	1	7.7
University of Pretoria	1	8.3	1	7.7
Did not specify	-	-	1	7.7
**Duration being an examiner (years)**				
1–5	5	41.7	-	-
5–9	2	16.7	-	-
> 9	5	41.7	-	-
**Participation in clinical examinations previously**				
Yes	11	91.7	-	-
No	1	8.3	-	-

SOE, structured oral examination.

The majority (*n* = 34, 85%) of candidates had been examined on three clinical cases, while 15% (*n* = 6) had to present five clinical cases. Twenty-eight (70%) of the candidates were attempting the examination for the first time. Of the remaining 30%, eight candidates had one previous attempt, and four had two.

For the qualitative study, two FGDs were conducted with examiners (10 examiners in total) and one FGD with four candidates. Examiners who participated were from six of the nine universities.

### Examiners’ and candidates’ experience of assessment of clinical competency

**FIGURE 1:**
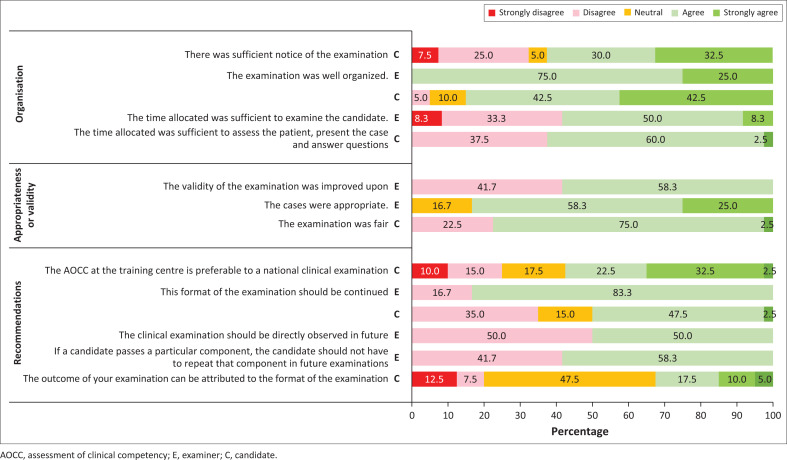
A divergent stacked graph depicting examiners’ and candidates’ views on the assessment of clinical competency.

The experiences of examiners and candidates in the AOCC were synthesised from their responses to the questionnaires (Figure 1) and from the FGDs. The next subsections integrate survey data with the supporting themes and quotes identified from the qualitative data.

#### Organisation and timing

While the majority (*n* = 25, 63%) of candidates who responded to the questionnaire felt they had sufficient notice of the examination, one candidate commented that they ought to have been notified of the examination dates ‘early enough’ to plan appropriately. Both examiners and candidates agreed that the examinations were well organised overall, with sufficient time allocated for the assessment (Figure 1). Examiners agreed that, given the AOCC’s objectives (i.e. to assess clinical competence), 15 min of examination time per candidate was sufficient.

#### Appropriateness, validity and fairness of the examination

Most examiners (58%) perceived that the validity of the examination had improved, and 83% considered the cases to be appropriate. Most candidates (*n* = 30, 76.9%) considered the AOCC to be fair (Figure 1). However, during the candidate FGD, a concern was raised regarding the reliability of the examination, for example:

‘… candidate x versus candidate y making the same mistake but having different outcomes in the examination.’ (FGD participant 3, candidate, female)

An examiner, who had previously participated in the CMSA examinations, expressed that certain examiners had developed reputations of being ‘hawks’ and that encountering them had a negative influence on candidates and their performance.

While candidates acknowledged that the format of the clinical examination had changed from two short cases and one long case to three short cases, they felt the examinations were of the same standard as before. A candidate mentioned that:

‘[*A*]lthough the long case was taken away and we just had the three short cases … I still felt like it was as intense as exams that I had ushered in previously.’ (FGD participant 1, candidate, female)

#### Decentralisation of clinical examination

Just over half of the candidates (*n* = 22, 55%) preferred a decentralised clinical examination over a national examination, and 47.5% (*n* = 19) endorsed the continuation of this format for future examinations, quoting cost and time saving as advantages. Candidates described the local examination as ‘less intimidating’ with a smaller number of examiners and candidates. They also perceived the examiners to be less stressed or frustrated.

However, with the local format, an examiner noted that there was a possibility that candidates may know the patients selected for the examination while working in the wards leading up to the clinical examination. Challenges were also raised regarding feedback and complaints, with a candidate pointing out the difficulty in ‘lodging complaints with the local examination as it is with current work colleagues’ (Questionnaire respondent 23, Candidate, male).

Certain centres conducted examinations for the first time during the pandemic. A candidate mentioned:

‘… smaller centres like [*X*] didn’t have enough FCP examiners, the examiners had to be outsourced from other institutions, that didn’t make the clinical competency much of a local exam.’ (Questionnaire respondent 10, candidate, male)

This was supported by an examiner who stated:

‘[*We*] had more logistics to arrange … But then it also meant [*our*] candidates were predominantly being examined by external examiners rather than examiners they were familiar with.’ (FGD 2 participant 3, examiner, male)

An additional concern raised by examiners was that the opportunity for faculty development might be limited in the local format. An examiner mentioned that the:

‘[*A*]rt of examining [*is*] a skill, it’s not something that everyone has immediately, most [*junior examiners*] really need to have an opportunity to examine with skilled examiners, so that [*they*] understand the art of examining.’ (FGD 2 participant 4, examiner, female)

While another examiner felt that ‘the art of medicine, and examination is lost in this form of examination’ (FGD 2 participant 2, examiner, male).

In the FGD, examiners agreed that the locally based AOCC examination was preferable from a logistical perspective. This format overcame many of the challenges of a national examination, including arranging examinations for a large number of candidates at a single centre. An examiner stated:

‘… regionalised exams are less logistically challenging [*as it makes it*] easier [*for*] a local organising team to be only arranging for four … or eight … or twelve candidates.’ (FGD 2 participant 3, examiner, male)

Another examiner mentioned:

‘… this format of the exam seems to make … organising [*the*] exam much more manageable and removes a lot of the anxiety and tension around the exam day.’ (FGD 1 participant 2, examiner, male)

Examiners also noted that examinations held in a local environment, where candidates are familiar with the disease profile, were less stressful than national examinations. One examiner noted that in the latter, you could always:

‘.… cut the tension in the air with a knife [*whereas*] just being in a familiar environment’ was perceived to contribute to ‘better candidate performance.’ (FGD 2 participant 3, examiner, male)

#### Format of clinical examination

Both examiners and candidates considered the number of cases in the AOCC as adequate. The additional two cases, when candidates were not successful in the first three, were considered to increase the validity of the examination. Candidates considered the inclusion of up to five clinical cases as advantageous. Short cases were preferred and described as ‘less stressful’ than the previous format, which included a long case. The local examination was seen as allowing a richer and ‘more holistic assessment of candidates as opposed to a once-off view of a candidate’ (Questionnaire respondent 33, candidate, male).

#### Examiners’ familiarity with candidates and role of external examiners

Differing opinions on the impact of examiners’ familiarity with candidates surfaced in the FGDs. It was perceived that examiners, from the same training centre as the candidates, may be influenced by the candidates’ perceived prior performance in a real-world clinical setting. Candidates felt that the potential for examiner bias is high with the local format, which can have both positive or negative impacts on the outcome for the candidate, depending on the examiner’s view of the candidate.

Another concern was the lack of external examiners, who could assist in moderation and objectivity, as would adherence to predetermined fixed objectives for each case and marking rubrics. Candidates also voiced concerns about the weight of expectations and pressure of ‘disappointing your examiners, who are the people who have shaped you through the four years’ (Questionnaire respondent 2, candidate, female).

#### Subjectivity and standardisation

Candidates felt that there was a lack of standardisation of training across institutions and commented that ‘some examiners from other institutions … ask questions on topics which were not emphasised in other institutions’ (Questionnaire respondent 22, candidate, female). Selection of cases was also raised by a candidate who mentioned that the cases candidates see on the day of the examination are the luck of the draw, and that if the candidate is having a bad day, it does not reflect their true ability. A suggestion made by a candidate was that ‘some form of continuous assessment should be in place from the onset of training’ (Questionnaire respondent 18, candidate, male). Examiners expressed concerns about a lack of uniformity in the allocation of the additional two cases. An examiner pointed out:

‘… the college [*CoP*] rules should be applicable to all universities that are training … because it may put other candidates at an extra advantage or disadvantage if it’s done differently.’ (FGD 2 participant 5, examiner, female)

#### Alignment of examination format to the role of a physician

Candidates reflected on whether the current format of the Part II examination resonated with the day-to-day functioning of a physician. A candidate stated:

‘I think that the current format of the exam is not suitable for how a person functions, either in the state as a physician or in private, because I think you’ll find that it’s still rooted in the olden days, where there was no technology, there was no investigation. So, I think that … the whole FCP [*examination*] is just something that is standalone and then there is the practice of medicine.’ (FGD participant 4, candidate, male)

Another candidate also highlighted the potential misalignment between the training received and examination format, stating that there is:

‘… too much time pressure to compose oneself and present accordingly. Training does not match the format of the examination.’ (Questionnaire respondent 28, candidate, male)

#### Disruptive preparation and reconfigured readiness

The pandemic and resultant changes in examination formats meant that candidates had to adjust their preparation. A significant challenge was ‘minimal bedside teaching during the COVID-19 pandemic’ (Questionnaire respondent 31, candidate, male). While candidates found the AOCC to be ‘very stressful’, it was ‘less stressful’ than travelling to another centre. The key advantage of the AOCC was that the environment and examiners were familiar to them. In the previous national examination, candidates from the hosting centre were perceived to have a ‘home ground advantage’. The local examination removed this variable. Other pandemic-related challenges included isolation from family and friends, the loss of loved ones, the inability to grieve normally because of restrictions on funerals and having to work through the pandemic, which significantly added to their overall examination-related stress. However, a candidate commended the university’s support in ‘accommodating’ candidates as they underwent this examination in ‘completely unforeseen circumstances’ (FGD participant 1, candidate, female).

### Examiners’ and candidates’ experience of structured oral examination

**FIGURE 2:**
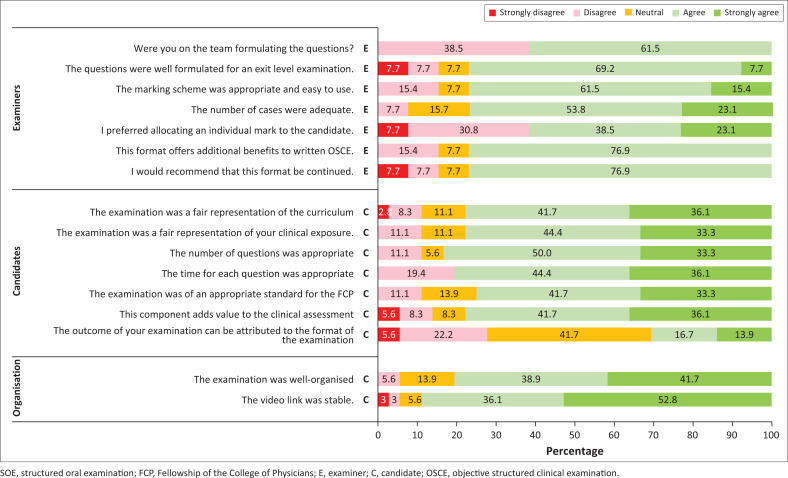
A divergent stacked graph depicting examiners’ (E) and candidates’ (C) views on the structured oral examination.

The participants’ experience of the SOE, from both the survey (Figure 2) and FGDs, is synthesised and discussed further.

#### Organisation of the structured oral examination

Most candidates found the SOE to be well organised and of a standard suited to the FCP(SA). In contrast, a candidate described the experience as ‘horrible’ because of the stress involved in the examination and the time between cases. However, they acknowledged the logistical challenges involved in coordinating an examination of this nature. Another candidate raised concerns about the late notification of the examinations, stating that there were ‘last minute announcements of upcoming exam dates (1 week before actual exam for both 2nd and 3rd component)’ (Questionnaire respondent 1, candidate, female). The overall duration was also a concern with a candidate stating:

‘[*T*]he exam duration is too long (and) exhausting. If there is way of combining the two exams, [*it*] would be much better.’ (Questionnaire respondent 8, candidate, female)

Another candidate suggested:

‘[*The*] written, clinical exam [*and*] SOE all should be done within four-weeks’ time, [*as*] doing exams over a four-month period is extremely stressful and tiring.’ (Questionnaire respondent 7, candidate, male)

These sentiments about the extended nature of the examination were expressed by other candidates as well, and were ‘worsened by long waiting periods for results’ (Questionnaire respondent 1, candidate, female).

While two-thirds (66.7%) of the candidates had a mock examination prior to the SOE, one candidate suggested:

‘… training sites [*should*] submit teaching plans to ensure preparation isn’t done as an afterthought by those tasked with the duty.’ (Questionnaire respondent 10, candidate, male)

Most of the examiners who responded to the questionnaire were on the team that formulated the questions for the SOE. The majority of them felt the questions were well formulated for an exit-level examination and that the number of cases was adequate.

#### Utility of the structured oral examination

Examiners’ opinions regarding the utility of the SOE varied. Some examiners felt that it enabled testing of a greater breadth of knowledge and complemented the AOCC, and its structured nature ensured greater objectivity. Furthermore, examiners could not fixate on a gap in the candidate’s knowledge and had to move on to the next question, enabling a fairer overall assessment. The online format also provided the ease of examining multiple candidates throughout the country without logistics and cost of travelling. While more than 70% of examiners recommended that this format be continued, one examiner stated that it still needs to be ascertained whether the SOE ‘adds anything to other exams – if it doesn’t it is not worth the effort’ (Questionnaire respondent 8, examiner, undisclosed gender). Examiners also raised a question regarding whether the SOE should be retained or what it should be replaced with, as an exit examination, if removed. The majority (77.8%) of candidates felt that the SOE component added value to the clinical assessment.

#### Tensions between standardisation and depth of clinical reasoning in the structured oral examination

Examiners thought that an advantage of the SOE was that it served as a national exit-level examination prior to candidates qualifying as physicians. Most examiners reported that the marking scheme was appropriate and user-friendly, and that they preferred allocating an individual mark. They also noted that the recording of SOEs allowed for review if required.

Candidates expressed that:

‘[*T*]he addition of [*the SOE*] is a good idea as it adds a bit of objectivity to the process, allows more topics to be assessed and provides a more all-round assessment.’ (Questionnaire respondent 16, candidate, male)

An examiner stated that there is:

‘ … uniformity across candidates as they have the same cases and same line of questioning.’ (Questionnaire respondent 6, examiner, undisclosed gender)

In contrast, a candidate noted:

‘… the elements tested in the SOE are already tested in the theory examination, therefore SOE questions could rather be incorporated into the written examination.’ (Questionnaire respondent 31, candidate, male)

The standardisation of questions and the recording of the virtual examination reassured the candidates that the SOE was a fair examination:

‘The examiner could not go on a tangent or delve into a subspecialty realm.’ (Questionnaire respondent 4, candidate, male)

The SOE was viewed as an opportunity to assess clinical approaches more effectively than in pressured clinical examinations, test a broader range of knowledge and enable standardisation of case scenarios across multiple sites per day. However, concern was expressed regarding the need to change cases on subsequent days, with one candidate stating:

‘it’s different questions for different candidates. Some candidates may be unfortunate to get more difficult scenarios.’ (Questionnaire respondent 18, candidate, male)

Candidates recommended further refinement and standardisation of the cases. However, not being able to interrogate or engage with candidates was seen as a disadvantage by examiners:

‘The fact that the candidate cannot be questioned or examined in more depth makes it difficult to ascertain the ability of clinical reasoning.’ (Questionnaire respondent 1, examiner, undisclosed gender)

Another examiner noted:

‘[*The SOE was*] impersonal and a bit uncomfortable for candidates - [*as there is*] no feedback.’ (Questionnaire respondent 10, examiner, undisclosed gender)

A similar view was expressed by a candidate as:

‘[*T*]here was no interaction in the 2020 exam. We were required to read a question and speak to a blank screen. Internal medicine is about discussion and [*a*] differential diagnosis. This format can be improved by making it a discussion around the questions with some feedback from the examiners …’ (Questionnaire respondent 11, candidate, female)

Similar comments included that there was a strange communication dynamic, that the nature of the examination was unidirectional and that there was no feedback from examiners if the candidate was on the right track. Other candidates felt that some questions were very open ended and could be interpreted in many ways, and that there was no way for examiners to redirect candidates. The lack of interaction while a video was recorded was seen as ‘even more intimidation’.

Candidate views on the structure varied, with one candidate feeling that there was ‘interference’ when asked additional questions beyond the predetermined questions, even though examiners were orientated beforehand, while another candidate found it ‘refreshing’ and appreciated the opportunity to clarify his answers.

#### Structure and content of the structured oral examination

An examiner suggested that candidates’ maturity in managing emergencies should be assessed and that there should be a good mixture of systems that commonly confront a physician in the real world.

Another examiner described the SOE as having:

‘… poorly constructed clinical cases with vague questions [*which is a*] disadvantage to those candidates who miss the essence of the clinical case and thus are lost for the whole question.’ (Questionnaire respondent 9, examiner, undisclosed gender)

The examiner advocated for a greater focus on evidence-based discussion:

‘[*T*]he actual outcomes tested for need to be refined [*so that it is*] not just repetition of theory, [*testing*] retention of knowledge, investigations and management.’ (Questionnaire respondent 9, examiner, undisclosed gender)

Furthermore, and examiner noted that there should be:

‘… consensus between examiners on what is expected from a candidate before commencing with the marking [*and*] perhaps a broader range for marking.’ (Questionnaire respondent 6, examiner, undisclosed gender)

Another examiner noted that inter-examiner variability should be assessed:

‘[*T*]he marks should be made available without much delay.’ (Questionnaire respondent 1, examiner, undisclosed gender)

#### Length of appointment of the convenor of the examination

An additional spontaneous suggestion from an examiner was that:

‘[*T*]he CoP should appoint a convenor and a team of physicians to manage the SOE for a period of 3–5 years and not allocate the exam to a different convenor each time. This will allow better continuity … It is very difficult for a new convenor to manage the exam.’ (Questionnaire respondent 3, examiner, undisclosed gender)

## Discussion

The COVID-19 pandemic catalysed unprecedented changes in medical education assessments worldwide. In this study, we report on the SA experience with the decentralised AOCC and digital SOE, reflecting both local innovation and global trends. While the descriptive findings of this study highlight the broad acceptability of both formats and cautiously recommend continuing them, a deeper critical analysis situates these adaptations within the wider discourse on assessment reform.

Comparative analysis reveals both convergence and divergence between SA and global trends. Like the UK’s remodelling of clinical stations and Canada’s phased CBME implementation,^4^ SA’s reforms were accelerated by the pandemic but rooted in pre-existing plans to change examination structure. The SOE’s emphasis on standardisation parallels international moves towards structured, competency-focused oral formats.

The feasibility of bedside clinical examinations, the bedrock of a physician’s assessment, was questioned in the face of the pandemic-driven circumstances.^10^ However, the CoP of SA decided to maintain real patient encounters, with the introduction of AOCC.^16^ SA’s adoption of decentralised bedside assessments contrasts with the simulation-heavy approaches in high-income contexts,^10^ reflecting both pragmatic adaptation and the enduring value placed on real patient interaction in resource-constrained settings.^18^

Reports from high-income countries documented widespread adoption of online and hybrid examinations during the pandemic, often prioritising fairness, safety and logistical feasibility over traditional validity measures.^10,11,12,13^

While the AOCC remains a summative examination, its decentralised delivery within local training environments shares selected characteristics with WBA, including increased contextual relevance, though this should not be construed as formal adoption of WBA principles.^19^ However, concerns about examiner familiarity and bias highlight the need for robust moderation and standardised rubrics to ensure fairness across sites.^20^

A recent South African study by Mrara et al.^20^ highlights how unconscious bias among examiners can influence outcomes in high-stakes postgraduate assessments, particularly oral and clinical examinations, reinforcing candidates’ concerns regarding fairness and equity in decentralised examination formats. These findings strengthen the argument for embedding structured WBAs within a CBME framework, where repeated formative assessments and external moderation may mitigate the impact of individual examiner bias and enhance the overall defensibility of assessment decisions.^19,20^

The shift also aligns with the growing emphasis on CBME and WBAs globally.^4,19^ Canada’s CBD model embeds continuous, formative assessments into training.^4^ In SA, the process of introducing WBA is well underway and has been initiated by several specialties.^18^ Advantages of WBA will be to increase the validity of assessments,^19^ overcome concerns over examiner bias and address candidates’ suggestions of other holistic aspects, such as astute use of investigations, writing adequate referral letters and discharge summaries and developing rapport between trainees and their peers, allied health workers and patients and their relatives.^18^

Remote oral examinations have emerged internationally as feasible alternatives to traditional in-person formats, and studies from SA and abroad demonstrate that web-based SOEs are both acceptable and logistically efficient, offering standardisation and broad content coverage.^10,14,15^

In the large mixed-methods study involving over 1400 candidates in SA, Burch et al.^14^ reported that most candidates found the web-based SOEs to be fair and effective in testing clinical reasoning, with most participants supporting their continued use. Our findings align with this study,^14^ highlighting the need for standardisation and objectivity in digital oral assessments. However, our study differs in several key respects. By focusing solely on the FCP examination and including both AOCC and SOE formats, we provide a more detailed analysis of the specific challenges and benefits within this specialty. Furthermore, our qualitative data reveal additional issues, such as examiner familiarity, potential bias in decentralised assessments and the impact of prolonged examination timelines. Our FGDs also surfaced recommendations for enhanced examiner training, external moderation and the integration of WBAs, offering practical guidance for future assessments. Thus, while our results corroborate the general acceptability of digital SOEs, they also underscore the importance of context-specific adaptations and continuous stakeholder engagement.

In the study by Lahri and Meyer,^15^ while participants recognised the value of SOEs for assessing clinical competence, concerns were raised about misalignment between examination content and clinical reasoning, heightened anxiety because of the high-stakes nature of the assessment and challenges faced by non-native English speakers. Like our study, examiners highlighted the need for targeted training in question design and bias mitigation to strengthen the effectiveness and fairness of SOEs. Furthermore, the authors advocated for the integration of WBA alongside SOEs, as suggested by candidates and examiners in our study as well.

The challenges of Internet connectivity and the stressful nature of the SOE expressed in our study mirror those expressed in Pakistan’s implementation of remote online assessments.^21^ Limitations such as reduced examiner–candidate interaction and uncertainty about the expected depth of responses have been consistently reported across settings.^10,11^ These concerns mirror those raised in our study, suggesting that while remote oral formats enhance objectivity and standardisation, they may diminish the nuanced interpersonal dynamics traditionally valued in oral examinations. Future models must therefore balance efficiency and accessibility with opportunities for probing clinical reasoning and fostering examiner–candidate dialogue.^10,15^

South Africa’s pandemic-driven adaptations were not merely reactive but represent a hybrid model that integrates global innovations with local realities.^22,23^ The AOCC and SOE demonstrate potential as sustainable formats, particularly if coupled with ongoing reforms towards CBME and WBA. Future research should interrogate how these formats can evolve into continuous, competency-based assessment systems that reduce reliance on single high-stakes examinations, thereby aligning SA more closely with international best practice while retaining contextual relevance.

Recognising patterns in the feedback could provide valuable insights for both the CMSA and educational institutions, ensuring that examinations remain rigorous, fair and reflective of the required competencies.

### Recommendations

In response to the central tensions identified in this study, including issues of standardisation, examiner bias and the limitations of high-stakes assessment, we propose the following recommendations for future assessment practice. While examiners and candidates broadly recognised the value of the AOCC in evaluating clinical competence, only a modest majority supported its retention in its current format. Based on persistent concerns regarding examiner familiarity and potential bias, we propose that external examiners should be appointed to ensure consistency. There should be standardisation across teaching platforms nationally, and training workshops should be conducted for the SOE.

Other recommendations include introducing mock examinations and continuous assessments, tailored to the AOCC format and targeted interventions for candidates who are not successful in the AOCC. Additionally, the interval between the three components of the examination should be shortened to reduce candidate anxiety and improve continuity. Furthermore, each component of the examination should be stand-alone; in that if a candidate passes a particular component, they should not be required to repeat it irrespective of their performance in other components.

The CMSA should engage more broadly with stakeholders regarding the conduct of examinations. The need to ensure congruency of registrar training with assessment models raised the question of whether there is still a place for a clinical examination or whether reconfiguration of the entire examination process is warranted.

### Strengths and limitations

By integrating a web-based survey and FGDs, our study provides nuanced, context-rich insights into the acceptability, perceived fairness and practical challenges of the revised assessment formats for the CoP and offers targeted recommendations for future reforms in physician assessment in SA.

However, there are several limitations. Firstly, the overall response rates were low, with only 15% of eligible candidates and 17.6% of examiners participating in the questionnaires, and the relatively small number of participants in the FGDs limits the generalisability of the findings. As the study was conducted during an evolving pandemic environment, this may have affected the response rate, and some participant reflections may have been influenced by rapidly changing circumstances and policies. Secondly, the voluntary nature of the study may have introduced sampling bias, as those with strong opinions – either positive or negative – may have been more inclined to respond. Thirdly, while FGDs added depth to the findings, the qualitative data may not fully represent the broader candidate or examiner populations, as there was under-representation of some training centres and potential group influences such as conformity or groupthink. This was partially mitigated by the inclusion of open-ended survey responses, which allowed participants to share their views confidentially. Fourthly, as with most survey-based research, the findings are subject to self-reporting bias, which may affect the accuracy or objectivity of participants’ responses, and may have been influenced by recall bias because of the timing of data collection and by response tendencies such as acquiescence or satisficing.

## Conclusion

During a period of great uncertainty, the decision by the CoP to introduce the AOCC and SOE was generally well received, offering important lessons for future competency-based assessments. Our study, despite its limitations, supports cautious retention and refinement of both the AOCC and SOE, while emphasising the need for increased standardisation, examiner training, external moderation and the phased integration of WBAs.

Importantly, the study contributes to the broader discourse on assessment reform by showing how SA’s adaptations both align with and diverge from global trends, offering lessons for competency-based and WBA models. In doing so, it provides evidence to guide the future evolution of physician examinations in SA and similar low- and middle-income contexts, ensuring that assessment practices remain valid, equitable and responsive to evolving healthcare and educational needs.

## References

[1] Membership of the Royal Colleges of Physicians of the United Kingdom. PACES 2020 [homepage on the Internet]. London: Royal College of Physicians of the United Kingdom; 2020 [cited 2020 Oct 06]. Available from: https://www.mrcpuk.org/PACES2020

[2] The Federation of the Royal Colleges of Physicians of the UK. MRCP(UK) Part 2 written examination [homepage on the Internet]. London: The Federation of the Royal Colleges of Physicians of the UK; [cited 2026 Jan 21]. Available from: https://www.thefederation.uk/examinations/part-2

[3] Royal College of Physicians and Surgeons of Canada (RCPSC). Competence by design [homepage on the Internet]. Ottawa, ON: RCPSC. [cited 2026 Jan 21]. Available from: https://www.royalcollege.ca/content/rcpsc-site-v2/ca/en/standards-and-accreditation/competence-by-design.html

[4] Frank JR, Snell L, Englander R, Holmboe ES, ICBME Collaborators. Competency-based medical education: Theory to practice. Med Teach. 2017;39(6):683–689. 10.1080/0142159X.2017.1315069 28598743

[5] Royal College of Physicians and Surgeons of Canada (RCPSC). CBD: In the meantime guide [homepage on the Internet]. 2016 [cited 2026 Jan 21]. Available from: https://medicine.usask.ca/documents/cbd/6RCPSCMeantimeGuide.pdf

[6] Van Der Vleuten C. The assessment of professional competence: Developments, research and practical implications. Adv Health Sci Educ. 1996;1(1):41–67. 10.1007/BF00596229 24178994

[7] Colleges of Medicine of South Africa (CMSA). Regulations for admission to the Fellowship of the College of Physicians of South Africa [homepage on the Internet]. Johannesburg: CMSA; 2019 [updated 2019 Mar; cited 2020 Oct 10]. Available from: https://cmsa.co.za/document/fcpsa-regulations/

[8] Nel D, Burch V, Adam S, et al. The introduction of competency-based medical education for postgraduate training in South Africa. S Afr Med J. 2022;112(9):742–743. 10.7196/SAMJ.2022.v112i9.16717 36214041

[9] Republic of South Africa. *Disaster Management Act (57/2002)*: Declaration of a National State of Disaster: COVID-19 (coronavirus) [homepage on the Internet]. Government gazette no. 43096, No. 313, 2020. [cited 2020 Oct 10] Available from: https://www.gov.za/sites/default/files/gcis_document/202003/43096gon313.pdf

[10] Boursicot K, Kemp S, Ong TH, et al. Conducting a high-stakes OSCE in a COVID-19 environment. MedEdPublish. 2020;9(1):54. 10.15694/mep.2020.000054.1 38058921 PMC10697458

[11] Sam AH, Millar KR, Lupton MGF. Digital clinical placement for medical students in response to COVID-19. Acad Med. 2020;95(8):1126. 10.1097/ACM.0000000000003431 PMC7179062 32744817

[12] Rose S. Medical student education in the time of COVID-19. JAMA. 2020;323(21):2131–2132. 10.1001/jama.2020.5227 32232420

[13] Sklar DP. COVID-19: Lessons from the disaster that can improve health professions education. Acad Med. 2020;95(11):1631–1633. 10.1097/ACM.0000000000003547 32544103 PMC7309647

[14] Burch V, McGuire J, Buch E, et al. Feasibility and acceptability of web-based structured oral examinations for postgraduate certification: Mixed methods preliminary evaluation. JMIR Form Res. 2024;8:e40868. 10.2196/40868 38064633 PMC10919348

[15] Lahri S, Meyer R. ‘We’re getting there’: Registrar and examiner perspectives on structured oral examinations in emergency medicine. J Coll Med S Afr. 2025;3(1):a206. 10.4102/jcmsa.v3i1.206 PMC12424041 40951605

[16] Colleges of Medicine of South Africa. FCP(SA) regulations [homepage on the Internet]. Colleges of Medicine of South Africa [cited 2026 Jul 15]. Available from: https://cmsa.co.za/document/fcpsa-regulations/

[17] Braun V, Clarke V. Using thematic analysis in psychology. Qual Res Psychol. 2006;3(2):77–101. 10.1191/1478088706qp063oa

[18] Ras T, Jenkins LS, Lazarus C, et al. ‘We just don’t have the resources’: Supervisor perspectives on introducing workplace-based assessments into medical specialist training in South Africa. BMC Med Educ. 2023;23:832. 10.1186/s12909-023-04840-x 37932732 PMC10629100

[19] Norcini J, Burch V. Workplace-based assessment as an educational tool: AMEE Guide No. 31. Med Teach. 2007;29(9–10):855–871. 10.1080/01421590701775453 18158655

[20] Mrara B, Oladimeji O, Rantloane AJ, Burch VC. Reducing unconscious bias in postgraduate specialist assessment in South Africa. Afr J Health Prof Educ. 2025;17(1):2–4. 10.7196/AJHPE.2025.v17i1.2089

[21] Ali S, Ali SMH, Saeed H, et al. Navigating the transition to remote online examinations in undergraduate medical education in a resource-limited setting: A student satisfaction and perception analysis. Cureus. 2025;17(5):e84480. 10.7759/cureus.84480 40539147 PMC12178450

[22] Baboolal SO, Singaram VS. Specialist training: workplace-based assessments impact on teaching, learning and feedback to support competency-based postgraduate programs. BMC Med Educ. 2023;23(1):941. 10.1186/s12909-023-04922-w 38082397 PMC10712152

[23] Daitz E, Jenkins LS, Janse van Rensburg J, Muller M, Singaram VS, Cooke R, et al. The introduction of workplace-based assessment into postgraduate medical training in South Africa: trainee perspectives. BMC Med Educ. 2026;26:515. 10.1186/s12909-026-08792-w 41723383 PMC13032551

